# Home ranges of raccoon dogs in managed and natural areas

**DOI:** 10.1371/journal.pone.0171805

**Published:** 2017-03-08

**Authors:** Karmen Süld, Urmas Saarma, Harri Valdmann

**Affiliations:** Department of Zoology, Institute of Ecology and Earth Sciences, University of Tartu, Tartu, Estonia; Centre for Cellular and Molecular Biology, INDIA

## Abstract

Knowledge of space use is central to understand animals’ role in ecosystems. The raccoon dog *Nyctereutes procyonoides* is considered as one of the most influential alien mesopredator species in Europe, having the potential to cause loss of local biodiversity and act as a vector for zoonotic diseases. We collared 12 animals to study their home range and habitat use in two areas with different management regimes in Estonia: in a protected natural area and in an intensively managed area. From May to October raccoon dogs inhabiting the natural area had considerably smaller home ranges compared to the managed area, 193.3ha±37.3SD and 391.9ha±292.9SD, respectively. This result contradicts somewhat earlier findings in other European raccoon dog populations, where the home range sizes in natural areas in summer and autumn period have usually been larger compared to managed areas. In both study areas raccoon dogs preferred watersides, where amphibians and other semi-aquatic prey are abundant, to other habitats available in their home ranges. We also studied movements of a raccoon dog pair in the managed study area in winter period. Due to mild weather conditions during the study period, raccoon dogs changed their resting sites quite often, covering a relatively large 599 ha area from November 2012 to January 2013, indicating the absence of usual winter lethargy during the mild winters.

## Introduction

Raccoon dog *Nyctereutes procyonoides* is an alien species in Europe, introduced from Far East of Russia to many regions in Eurasia since 1929 and to the current territory of the European Union since 1947 [1,2]. The species is now well-established in Europe and continues to expand its range towards the west and south of the continent [3]. The raccoon dog is considered among the most invasive alien species in Europe [4], having the potential to cause loss of native biodiversity, but also economic and health problems [5]. There have been concerns about the negative impact of the raccoon dog to ground nesting birds and amphibians [6–8], but according to Kauhala and Auniola [9] the negative effect can be significant primarily at local scales (e.g. in shores and small islands). In Europe, along with the red fox (*Vulpes vulpes*), raccoon dog is one of the main vectors of zoonotic diseases such as rabies, alveolar echinococcosis and sarcoptic mange, the first two being highly hazardous also to human health [10].

The raccoon dog was introduced to Estonia in 1950, although the species occurrence was reported already in 1938 [11], and is currently distributed all over the country, including most of the islands and seasonally also the islets close to mainland. Its densities have fluctuated significantly over the past decades due to changes in hunting pressure, depending largely on fur prices, but also due to a recent successful vaccination campaign against rabies. The species population size in Estonia is unknown and therefore the total harvest rate is the only proxy for population trends. The number of hunted individuals has increased from about four thousand in 2005, which was also the first year of the vaccination campaign, to over thirteen thousand in 2013 [12] when Estonia was declared to be rabies free [13]. However, the significant increase in population size, and not only of raccoon dogs but also of red foxes, brought about an extensive spread of sarcoptic mange, that is caused by the ectoparasitic itch mite *Sarcoptes scabiei*. As a result, the hunting statistics as well as observations by hunters have indicated noticeable decrease in population size during past two years [12]. Recently, another zoonotic pathogen has been discovered in Estonia, namely the fox tapeworm (*Echinococcus multilocularis*), causing alveolar echinococcosis. The parasite was first detected in red foxes, affecting about one third of the wild population [14] and about 7% of foxes in urban area [15], and recently also in raccoon dogs, albeit with a significantly smaller portion (1.6%) of infected individuals [16].

Since the raccoon dog is capable of affecting local ecosystems and is a vector for a number of hazardous zoonotic diseases in Estonia [17], it is important to understand the species home range and habitat selection. According to studies performed in Finland, Poland and Germany, raccoon dog home range size varies from 50 ha to 810 ha, in general being smaller in managed areas compared to natural environment [18–21]. The size of area used by raccoon dogs mainly depends on season, landscape, available habitats, food sources and human impact. For example, raccoon dogs tend to have significantly smaller home ranges in areas where the landscape is mosaic, providing both natural as well as managed habitat patches (gardens, fields), opposed to areas dominated by large monocultural fields or forest arrays [20–26].

The main objective of this study was to investigate the raccoon dog home range size and habitat use in areas with different management regimes in Estonia: in an intensively managed area with anthropogenic influence and in a protected natural area. Taking into account the data from studies carried out elsewhere in Europe, we predicted the home ranges of raccoon dogs to be smaller in managed areas and larger in a protected natural area.

## Materials and methods

### Ethics statement

All procedures concerning the capture and handling of wild animals for scientific purposes in the nature are regulated by the Estonian Ministry of Environment that issued a permit (no 1–4.1/11/146) for this study. The capture and handling was coordinated with the local department of the Estonian Ministry of Environment and hunting district of Tähtvere (Tartu County, Estonia).

Three of the study animals were captured by a specially trained hound (German Wirehaired Pointer) of a local hunter. The hound located and cornered the raccoon dogs without biting them. Nine raccoon dogs were captured with wire box traps, which were controlled once or twice a day, to avoid dehydration of the animals.

As raccoon dogs are easy to handle, due to characteristic demeanour of the species to play dead in threat situations, the captured animals were collared without administrating any anaesthetics. Handling the animals without immobilizing them with drugs helps to avoid any physical distress related to recovery process. During collaring process, the raccoon dogs were kept still by a skilled specialist until the other person adjusted the collar. The collars were fitted loosely enough to avoid possible discomfort caused by thickening of the neck during periods of intensive feeding and fat accumulation. To be sure that the raccoon dogs had recovered from the stressful collaring process, all animals were observed until they left the place of capture.

Six of the study animals were suited with VHF-radio collars and six animals with GPS-collars, which weighed 75 g and 210 g respectively, constituting less than five percent of raccoon dogs’ average body weight.

### Study areas

**Fig 1:**
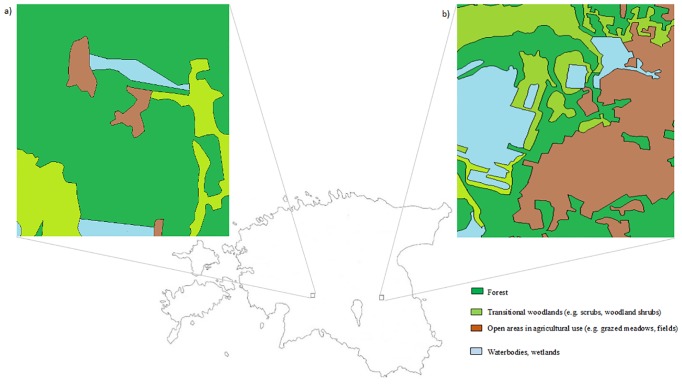
Locations of study areas in Estonia. a) protected natural area in Soomaa National Park (78 ha; 58°25´N, 25°1´E); b) intensively managed area nearby Ilmatsalu borough (103 ha; 58°2´N, 26°32´E).

The study was carried out in two different areas: a) in a protected natural area in Soomaa National Park in central Estonia (78 ha; 58°25´N, 25°1´E), and b) in an intensively managed area in southern Estonia nearby Ilmatsalu borough (103 ha; 58°24´N, 26°32´E). Life of raccoon dogs and other animals in Soomaa is strongly affected by changes in water regime. In springtime, vast amount of water runs down the Sakala Upland, covering grasslands and forests and forming the Riisa flood area with a size of more than one hundred square kilometres. Extensive flooding is also common in autumn. There are numerous abandoned households scattered throughout the study area and only one functional household in the centre of the area. Regular grass mowing and grazing by highland cattle in small patches of meadows are the only practiced agricultural management form. Altogether 81.4% of Soomaa study area is covered with forests, 10.2% with scrubs and woodland shrubs, 4.6% with managed meadows and 3.8% wetlands and water bodies (reed beds, rivers, drainages ditches, ponds) (Fig 1). In contrast, the Ilmatsalu study area with the population density of 22 residents per square kilometre is characterized by more extensive anthropogenic activities: small farmsteads cover about 2.1% of the area and altogether 32.9% is used for agricultural purposes, while the remaining of the area is covered with forests (38.8%) and transitional woodlands (scrubs and woodland shrubs; 19.7%) (Fig 1). The area is also part of a local hunting ground. The only types of water bodies in the area are drainage ditches and fish ponds (6.6%), but flooding is characteristic also to this area in springtime. The average annual temperature during the study period was 5.9°C in Soomaa and 5.3°C in Ilmatsalu area, with total annual precipitation of 835.5 mm and 771.5 mm, respectively (The Estonian Environment Information Centre). Both study areas are inhabited by a diverse community of carnivores: in addition to the raccoon dog also by red fox, gray wolf (*Canis lupus*), brown bear (*Ursus arctos*), European lynx (*Lynx lynx*), Eurasian badger (*Meles meles*) and other mustelids (*Mustelidae*). During our study there was a severe outbreak of sarcoptic mange in Ilmatsalu study area.

### Radio-tracking

In Soomaa study area the raccoon dogs (n = 3) were caught by using specially trained hounds and the radio-tracking was carried out from May to October 2009. In Ilmatsalu area, where raccoon dogs (n = 9) were captured with wire box traps, the study was conducted from March 2012 to November 2013. Captured animals were collared without drug immobilisation as raccoon dogs are easy to handle without [23,27]. It was determined by visual and physical examination that all the animals were adults. All raccoon dogs from Soomaa and three from Ilmatsalu study area were suited with Telonics model 210 VHF-radio collars. Two raccoon dogs from Ilmatsalu area with VHF-collars formed a pair (M6_vhf_IL and F5_vhf_ILM, see Table 1). The remaining six raccoon dogs from Ilmatsalu were suited with MiniTrack210 GPS-collars (LOTEK Wireless Inc.). One animal from Ilmatsalu had symptoms of sarcoptic mange at the time of collaring and due to limited data from that animal (M3_gps_IL, see Table 1), it was excluded from statistical analysis.

**Table 1:** Sizes of 95% fixed kernel home ranges (K95) and 100% minimum convex polygon (MCP100) areas in hectares for each radio-tracked raccoon dog in Soomaa and in Ilmatsalu study area. Note that kernel home ranges were not calculated when there were less than 50 location point available for the animal.

Raccoon dog ID	No of data points	Summer		Autumn		Total study period	
		K95	MCP100	K95	MCP100	K95	MCP100
**F1_gps_IL**	750	227	788				
**M1_gps_IL**	650	108	276				
**F2_gps_IL**	299			73	143		
**F3_gps_IL**^b^	44				554		
**M2_gps_IL**	110			272	830		
**M3_gps_IL**^b^	5				80		
**F4_vhf_SM**	198	138	188	73	109	155	236
**M4_vhf_SM**	119	57	78	135	161	112	177
**M5_vhf_SM**	135	136	165		64	112	167
**M6_vhf_IL**^a^	71		179		275		207
**F5_vhf_IL**^a^	67		189		211		257

F—female, M—male; gps—with GPS collar, vhf—with VHF collar; IL—Ilmatsalu study area, SM—Soomaa study area; N—number of location points for animal.

^a^ Raccoon dogs M6_vhf_IL and F5_vhf_IL formed a pair.

^b^ Data of raccoon dogs F3_gps_IL and M3_gps_IL were excluded from further analysis.

Raccoon dogs with VHF-collars were located with a handheld four-element Yagi-antenna (Y-4FL, Televilt, TVP) and with Telonics TR-4 receiver. For animals with GPS-collars, three-element flexible Yagi-antenna (AN-3FX 172), SIKA receiver (SIKARX4) and handheld VHF Command Unit (GPS-CMD VHF) were used. Tracking sessions were carried out (VHF-collars) or scheduled (GPS-collars) from 6 pm to 6 am when raccoon dogs are most active [25,27] (also our own observations). For raccoon dogs with VHF-collars triangulation method was used to take the bearings, keeping the angle between bearings close to 90°. To avoid measurement errors due to animal movements, bearings were taken within as short time period as possible (5±2 minutes). The minimal time interval between triangulations was 25 minutes and the location error was measured to be about 100 m (103±55.8SD) by locating hidden VHF radio-collars (35 location points).

We divided the study period into three different seasons: spring (March-April) as a mating season, summer (May-July) as a time of cub rearing; autumn (August-October), the period of intensive foraging and fat accumulation; and winter (November-January) as a period of reduced activity due to cold weather. Coordinates for winter locations were obtained only for two raccoon dogs forming a pair in Ilmatsalu area by seeking out sleeping sites of the animals at least once a week.

### Home range calculations and habitat analysis

Home range areas were calculated for each animal by using the fixed kernel method and minimum convex polygon method (MCP). For kernel estimations we used the least squares cross validation smoothing parameter. We considered 95% kernel home range (K95) as the total home range area as the 100% kernels tend to overestimate the actual home range size due to occasional trips outside the most frequently used area [28]. The 95% kernel (K95) home ranges were not calculated when there were less 50 location points available for the animal [29]. The 100% minimum convex polygons (MCP100) were calculated for the purpose of comparison with previous home range studies (e.g. 19; 21), as MCP method has been the most widely used method.

Compositional analysis method [30] was used to study habitat use and selection. We obtained relative habitat use for fixes within home ranges. Availability of different habitat types was estimated by using CORINE Land Cover 2006 map. We used Jacobs’ selectivity index *P*_*i*_ = *(U—V)/(U+V–2UV)*, where *U* is the proportion of habitat used and *V* the proportion of habitat available [31], to estimate habitat preferences within study areas. The *P*_*i*_ values can vary from -1 to +1. Values below 0 indicate preference, values over 0 indicate avoidance and values equal or close to 0 indicate that habitats are used in proportion to their availability.

Home range analysis was conducted with RANGES 8. Programs Biotas and R (Package ‘adehabitatHS’) were used for habitat analysis. The data (coordinates of location points) used for analysis is available in Table A in S1 File.

## Results

### Home ranges sizes and habitat use

In summer and autumn periods, the raccoon dogs in Ilmatsalu used considerably larger (~2x) areas than in Soomaa, the mean MCP100 home range sizes of all analysed individuals being 391.9ha±292.9SD and 193.3ha±37.3SD, respectively (Table 1). In both study areas the summer home ranges (MCP100) were significantly larger than autumn ranges (Soomaa: 143 ha vs 111 ha; chi-square = 83.426, df = 2, p<0.001; Ilmatsalu: 358 ha vs 243 ha; chi-square = 991.3, df = 4, p <0.001); only home ranges with over 50 location points per season were included on the seasonal comparison. In winter period raccoon dog pair from Ilmatsalu changed their resting sites (n = 18) in the area covering 599 ha (MCP100). For one raccoon dog from Ilmatsalu we had only 30 location fixes from the time period March to April 2012 and the data was excluded from general analysis. The total home range size for that animal was 72 ha (MCP100).

Although in both study areas most of the location points could be found in forest habitat, watersides were used more often than could be expected from the proportion of availability of the habitat (Table 2). Also, according to preference indices (*P*_*i*_), in Ilmatsalu study area raccoon dogs distinctively preferred watersides (*P*_i_ = 0.77) to other habitats (open area *P*_*i*_ = -0.34; forests *P*_*i*_ = -0.22; shrubs *P*_*i*_ = -0.2). While P_i_ value for watersides was also the highest (0.38) compared to other habitats in Soomaa, the preference was not as pronounced as in Ilmatsalu (shrubs *P*_*i*_ = 0.36; open areas forests *P*_*i*_ = -0.2; forests *P*_*i*_ = -0.38).

**Table 2:** The proportion (%) of different habitat types in Soomaa and Ilmatsalu study areas (Avail) and in 95% fixed kernel home ranges (K95) of raccoon dogs radio-tracked in summer and autumn period.

Habitat type	Soomaa			Ilmatsalu		
	Avail	Summer	Autumn	Avail	Summer	Autumn
Open area	4.5	3.5	33.3	35.0	21.0	25.4
Forests	81.8	71.6	47.4	38.8	34.7	36.0
Shrubs	10.0	15.3	8.8	19.6	10.2	19.1
Watersides	3.7	9.6	10.5	6.6	34.1	19.5

In autumn period raccoon dogs could be quite often located in meadows and fields (open area). Nonetheless, no significant habitat selection could be found (summer period: ʎ = 0.59, *χ*^2^ = 0.917, p = 0.296; autumn period: ʎ = 0.77, *χ*^2^ = 0.454, p = 0.558).

## Discussion

Although based on a relatively small sample size, our study results indicate that home ranges of raccoon dogs could be considerably smaller in forest-dominated areas, where the human impact to natural environment is minimal compared to the intensively managed areas. We expected the opposite, as studies conducted elsewhere in Europe have shown that home ranges of raccoon dogs are generally smaller in landscapes where majority of the land is in agricultural use and intensively managed compared to areas dominated by forest and other natural habitats [18,21,24,25] (see also Table 2). However, studies from Finland and Germany have also demonstrated that size of the area used by a raccoon dog depends on habitat richness: raccoon dogs tend to have smaller home ranges in areas with a variety of small habitat patches [18,20,23,26] (see also Table 2). For example, Kauhala et al. [26] showed that in Finland in areas dominated by spruce forest, home ranges of raccoon dogs were twice the size compared to the areas comprised of meadows, mixed forests and gardens. The negative impact of small habitat patches to the home range size of the raccoon dog was also evident in the study of Drygala et al. [22] in north-eastern Germany, but not in the study conducted in the same region by Sutor and Schwarz [21]. Although both investigations took place in managed areas, home ranges were considerably smaller in the study area described in the latter study, which had higher diversity of habitat types—forests with abandoned badger dens, hedges, and grasslands—compared to the former, which was characterised by vast homogenous crop fields [21,22] (see Table 2). Similar pattern to that observed by Sutor and Schwarz [21] was characteristic also to our study, as the range of different habitat patches was more diverse in Soomaa than in Ilmatsalu. The habitat type ‘forest’ consisted of deciduous, coniferous and mixed forests in Soomaa, while only of mixed forest in the Ilmatsalu study area. In addition, while the drainage ditches were the only type of water bodies represented within the home ranges of the raccoon dogs in Ilmatsalu, two rivers flowing through the Soomaa study area provided additional habitats such as reed beds and meadows. Therefore, it is possible, that at a smaller scale the habitat composition in Soomaa was more suitable for raccoon dogs than in Ilmatsalu.

However, we cannot overlook the impact of diseases and hunting pressure when comparing Soomaa and Ilmatsalu study areas. During the study period there was an outbreak of sarcoptic mange in Ilmatsalu area, which kept the population density low, allowing for the healthy raccoon dogs to broaden their home ranges. Moreover, the hunting pressure during the study period was high in Ilmatsalu area (over 50 kills per 12 000 ha; personal communications with local hunters). Hence it is likely that the population density of the raccoon dogs was considerably higher in Soomaa study area, where any form of game management was forbidden and no evidence of spread of sarcoptic mange could be noticed during the study period (pers. com. with Soomaa National Park managers; our own observations).

It is common for raccoon dogs to broaden their home ranges in autumn compared to summer, as the cub rearing period has ended and raccoon dogs start to accumulate fat reserves for the coming winter period [21,22,32]. It is interesting to note that in Ilmatsalu area, the raccoon dog pair radio-tracked through both seasons, enlarged their movement area in autumn, whereas two animals from Soomaa reduced their home ranges by half during the same period (Table 1). This seemingly contradictory behaviour could be explained by the changes in the water level in Soomaa. Just like in spring, the flooding occurs also in autumn, restricting movements of raccoon dogs on the overflown water meadows surrounding the rivers. The other reason for smaller autumn home ranges could be an increased availability of anthropogenic food in this period. One of the raccoon dogs shifted its range to the vicinity of the Soomaa National Park Centre where ripe fruits and food waste from the compost heap were available. The other raccoon dog visited repeatedly at least two old orchards (relicts of the abandoned farmsteads) where apples were readily available. Though households with orchards existed also within and nearby the home range of the raccoon dog pair from Ilmatsalu, those were fenced and guarded by dogs, making the potential food sources inaccessible. The latter might have also affected the home range size, forcing the raccoon dog pair to forage in larger areas.

As for the habitat use in the summer period, raccoon dogs in both study sites most often roamed in forest areas (Table 2), which provide safe environment (burrows, as well as food) for the period of pup rearing. Still, throughout the study period, raccoon dogs used forest habitats in proportion to their availability and showed clear preference only towards watersides. In autumn, the raccoon dogs were more frequently found on meadows, in shrubs and in reed beds at riversides. This kind of seasonal shift in habitat selection could be explained by phenological changes in food availability, e.g. the amphibians gather nearby the watersides during their mass migrations, making themselves easily accessible prey for the opportunistic raccoon dog. Indeed, in Estonia the frequency of occurrence of different kind of plant material (FO = 89.9%) and also amphibians (FO = 14.4%) in raccoon dog diet is rather high in autumn period [33]. Also, after pups become independent and start to disperse, adult raccoon dogs expand their home ranges to forage more intensively and accumulate fat reserves before winter. Compared to early spring, raccoon dogs double their body weight by the end of autumn (4.3 kg *vs* 8.6 kg) [34].

**Fig 2:**
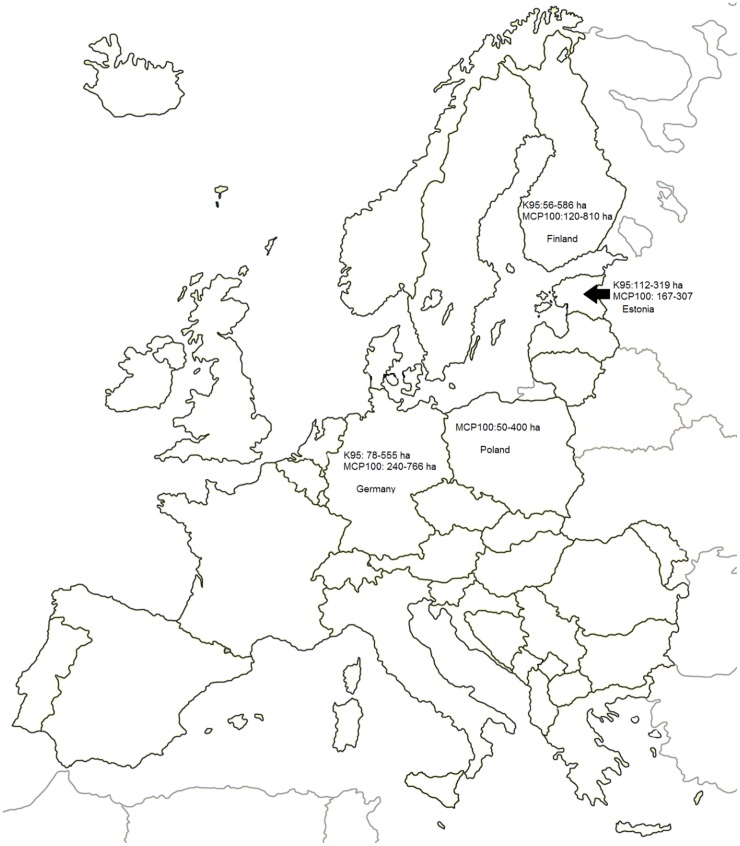
Home range sizes of raccoon dogs from different areas in Europe. K95–95% kernel home range; MCP100–100% minimum convex polygon; ha—hectares. Estonia—this study. Finland—Holmala and Kauhala 2009; Kauhala and Auttila 2010; Kauhala et al. 2010; Kauhala and Holmala 2008; Kauhala et al. 1993. Mustonen et al. 2012; Poland—Jedrzejewska and Jedrzejewski 1998. Germany—Drygala and Zoller 2013; Sutor and Schwarz 2012; Drygala et al. 2008a.

We also studied movements of the raccoon dog pair in Ilmatsalu area from November 2012 to January 2013. The pair stayed together for the whole study period and moved around extensively, covering the area of 599 ha. As this result is based on only 18 resting sites it is possible, that the area where the raccoon dogs moved around could have been even larger. Still, our results reported here are similar to the winter home range reported in eastern Finland (586 ha) [35] (see also Fig 2, Table B in S1 File). In Poland [19], the movements at this period ranged from 50–400 ha. In Germany, where weather conditions are milder in winter, home ranges size have reached up 669 ha [22] (see also Fig 2, Table B in S1 File). Larger home range sizes in winter period could be explained by the need to cover long distances between suitable resting and feeding sites. In our study area, most of the resting sites were situated in reed beds on and around the ponds, which were drained for the winter period. The preference of open areas in winter period has also been demonstrated by Mustonen et al. [35].

Kauhala et al. [27] have shown that raccoon dogs stay dormant in their dens when air temperature is below -10°C and snow depth is over 35 cm. In the Ilmatsalu study area, Oja [36] has registered the absence of raccoon dogs at feeding places in Ilmatsalu at temperatures below -6°C. The frequent changes of the resting sites observed in Ilmatsalu area were probably due to mild weather conditions: during the study period the snow depth did not exceed 16 cm, and the short periods when temperature dropped below -10°C quickly alternated with the thaw (Estonian Weather Service). Kauhala et al. [27] and Mustonen et al. [35] have pointed out that frequent movements of raccoon dogs during their usual winter lethargy could facilitate the spread of diseases. During the study period there was an outbreak of sarcoptic mange in the Ilmatsalu study area and this was the likely cause of death of the raccoon dog pair in March 2013, although both animals were in good condition when observed in January.

## Supporting information

S1 File **Table A** Coordinates of location points used for data analysis. **Table B** Seasonal home range sizes of raccoon dogs in different areas in Europe. Home range sizes correspond either to average values or to the range from minimum to maximum if two values are given.
(DOCX)
