# Supplementary material for: Home ranges of raccoon dogs in managed and natural areas
Source: PLoS One. 2017 Mar 8;12(3):e0171805. doi: 10.1371/journal.pone.0171805 (PMC5342178; doi:10.1371/journal.pone.0171805)
Supplement: S1 File — Table A Coordinates of location points used for data analysis. Table B Seasonal home range sizes of raccoon dogs in different areas in Europe. Home range sizes correspond either to average values or to the range from minimum to maximum if two values are given. (DOCX) [file pone.0171805.s001.docx]

| **Study area** | **N** | **Year** | **Season** | **K95/MCP100 (ha)** | **Reference** |
| --- | --- | --- | --- | --- | --- |
| Estonia | 6 | 2009-2013 | Spring  Summer  Autumn  Winter | 116/72  57-226/78-189  66-360/64-275  -/599 | This study |
| Eastern Finland | | | | | |
| Kaatamo, Ristinkyla | 14 | 2006-2007 | Winter | 56-586/- | Mustonen et al. 2012 |
| Southern Finland | | | | | |
| Ruissalo, Tuulos | 36 | 2005-2008 | Summer | 99-104/- | Kauhala and Auttila 2010 |
| Ruissalo, Tuulos, Virolahti | 42 | 2001-2007 | Summer | 93-261/130-312 | Kauhala et al. 2010 |
| Virolahti | 18 | 2000-2004 | Summer, autumn | 321/- | Holmala and Kauhala 2009 |
| Virolahti | 14 | 2001-2003 | Summer, autumn | 299-343/- | Kauhala and Holmala 2008 |
| Evo | 19 | 1989-1991 | Summer  Autumn | -/520-690  -/780-810 | Kauhala et al. 1993 |
| Poland (Bialowieza) | - | - | Winter | -/50-400 | Jedrzejewska and Jedrzejewski 1998 |
| Northeastern Germany | | | | | |
| Meckelenburg-Western Pomerania | 13 | 2004-2006 | Spring  Summer  Autumn  Winter | 150/-  112/-  124/-  78/- | Drygala and Zoller 2013 |
| Brandenburg | 12 | 2001-2004 | Spring  Summer  Autumn  Winter | 206/-  180/-  237/-  85/- | Sutor and Schwarz 2012 |
| Meckelenburg-Western Pomerania | 26 | 1999-2003 | Spring  Summer  Autumn  Winter | 158-159/284-314  194-225/240-444  528-555/706-766  283-291/630-669 | Drygala et al. 2008a |

**Table S1** Seasonal home range sizes of raccoon dogs in different areas in Europe. Home range sizes correspond to either average values or to the range from minimum to maximum if two values are given.
